# The relationship between plasminogen activator inhibitor-1 and type 2 diabetes: a systematic review and meta-analysis

**DOI:** 10.1186/1758-5996-7-S1-A119

**Published:** 2015-11-11

**Authors:** James Yarmolinsky, Natália Bordin Barbieri, Tobias Weinmann, Bruce B Duncan, Maria Inês Schmidt

**Affiliations:** 1 UFRGS, Sapiranga, Brazil

## Background

An emerging body of evidence has implicated plasminogen activator inhibitor-1 (PAI-1), a key regulator of the fibrinolytic system, in the development of type 2 diabetes (T2D), though findings have not always been consistent.

## Objectives

We systematically reviewed epidemiological studies examining the association of PAI-1 with T2D.

## Materials and methods

EMBASE, PubMed, Web of Science, and the Cochrane Library were searched by two independent reviewers for all relevant studies published from 1945 to October 2014. Studies were included if they met all of the following inclusion criteria: 1) Prospective or retrospective cohort study, case-cohort, case-control design, or cross-sectional study; 2) Measurement of plasma PAI-1 (antigen concentrations or activity levels); 3) Assessment of T2D (self-reported physician diagnosis and/or medication usage and/or laboratory diagnosed); 4) Adult study population (≥18 yrs.) at baseline, and 5) Article was reported in English. Prospective studies were pooled using a random-effects model to generate summary RRs and 95% CIs.

## Results

49 studies (41 cross-sectional with 44 unique analytical comparisons and 8 prospective) were included. Out of 44 cross-sectional comparisons, 33 (75%) reported significantly elevated PAI-1 among diabetes cases versus controls, 2 (5%) reported significantly elevated PAI-1 among controls, and 9 (20%) reported null effects. In pooled random-effects analyses of prospective studies including 8612 participants and 932 incident diabetes cases, a comparison of the top third vs. bottom third of baseline PAI-1 values generated a RR of T2D of 1.68 (95% CI 1.29-2.18) with moderate heterogeneity (I2=37%). These results did not differ substantially by study design (prospective cohort or nested case-control), length of follow-up (≥ or < 5.7 yrs.), or adjustment for measures of insulin, glucose, visceral adiposity, or inflammatory markers, and were robust to sensitivity analyses. Visual inspection of funnel plots and Results from Begg's and Egger's tests suggested absence of publication bias.

## Conclusions

Findings from this systematic review of the available epidemiological literature support a link between PAI-1 and T2D. Pooled effects from 8 prospective studies suggest that elevated PAI-1 levels predict incident T2D, independently of established diabetes risk factors. Given the moderate size of the association and heterogeneity across studies, future prospective studies are warranted.

